# Age-Related Hearing Loss: The Link between Inflammaging, Immunosenescence, and Gut Dysbiosis

**DOI:** 10.3390/ijms23137348

**Published:** 2022-07-01

**Authors:** Dagmara Kociszewska, Srdjan Vlajkovic

**Affiliations:** Department of Physiology and The Eisdell Moore Centre, Faculty of Medical and Health Sciences, The University of Auckland, Private Bag, Auckland 1142, New Zealand; d.kociszewska@auckland.ac.nz

**Keywords:** age-related hearing loss, presbyacusis, immunosenescence, inflammation, gut dysbiosis

## Abstract

This article provides a theoretical overview of the association between age-related hearing loss (ARHL), immune system ageing (immunosenescence), and chronic inflammation. ARHL, or presbyacusis, is the most common sensory disability that significantly reduces the quality of life and has a high economic impact. This disorder is linked to genetic risk factors but is also influenced by a lifelong cumulative effect of environmental stressors, such as noise, otological diseases, or ototoxic drugs. Age-related hearing loss and other age-related disorders share common mechanisms which often converge on low-grade chronic inflammation known as “inflammaging”. Various stimuli can sustain inflammaging, including pathogens, cell debris, nutrients, and gut microbiota. As a result of ageing, the immune system can become defective, leading to the accumulation of unresolved inflammatory processes in the body. Gut microbiota plays a central role in inflammaging because it can release inflammatory mediators and crosstalk with other organ systems. A proinflammatory gut environment associated with ageing could result in a leaky gut and the translocation of bacterial metabolites and inflammatory mediators to distant organs via the systemic circulation. Here, we postulate that inflammaging, as a result of immunosenescence and gut dysbiosis, accelerates age-related cochlear degeneration, contributing to the development of ARHL. Age-dependent gut dysbiosis was included as a hypothetical link that should receive more attention in future studies.

## 1. Introduction

Age-related hearing loss (ARHL), or presbyacusis, is a type of sensorineural hearing loss that primarily affects the elderly [1]. However, the age of onset, rate of decline, and severity of hearing loss vary widely.

ARHL is the most common sensory disorder, with a high economic impact [2,3]. The World Health Organization (WHO) estimates that by 2050, 2.5 billion people, predominantly over 60, will be living with some degree of hearing loss [4,5]. Despite the high prevalence of this sensory disorder, there is a paucity of both preventative and treatment strategies other than prosthetic devices (hearing aids and cochlear implants). 

Presbyacusis typically presents as bilateral, progressive, and irreversible [6,7]. The increasing prevalence of presbyacusis may be attributable to environmental factors, notably noise exposure and the rise in metabolic diseases [8,9,10].

This sensory disorder can be characterised by reduced hearing sensitivity and speech understanding in background noise, slowed central processing of acoustic information, and impaired localisation of sound sources [7]. Hearing loss affects high frequencies initially and eventually spreads to lower frequencies involved in speech understanding [7]. Untreated hearing impairment contributes to social isolation, loss of self-esteem, depression, and cognitive decline [11,12,13]. Even mild levels of hearing loss increase the long-term risk of cognitive decline and dementia [14].

ARHL has a complex pathophysiology linked to genetic risk factors that determine the rate and extent of cochlear degeneration. However, the severity of the hearing loss is also influenced by previous otological diseases, chronic illnesses, cumulative noise exposure, use of ototoxic drugs, and lifestyle [15]. Moreover, this condition has been associated with numerous comorbidities, including dementia, frailty, Alzheimer’s disease, and type II diabetes [16,17,18,19,20]. A common trait of these disorders is chronic inflammation in target organs [21]. More recently, changes in gut microbiota have been linked to systemic inflammation affecting multiple organ systems, including the brain and the inner ear [22,23,24]. This review focuses on cochlear inflammation, immunosenescence, and gut dysbiosis as putative underlying factors of ARHL.

## 2. Methodology

A literature search of related publications from 1995 to 2021 was conducted using PubMed, Google Scholar, and Embase medical databases. In addition, reference lists from the relevant papers were used. Following Boolean search logic, the main keywords included were: (age-related hearing loss OR presbyacusis OR cochlea) and (diet OR obesity OR diabetes OR diet-induced obesity OR inflammation) and (microbiota OR gut dysbiosis OR reactive oxygen species OR lipopolysaccharide OR high-fat diet OR intestinal hyperpermeability OR inflammatory bowel disorder OR inflammaging OR immunosenescence OR neurodegeneration or inflammation). The search results were consequently examined according to their relevance to this review. Only English language publications were included.

## 3. Pathophysiology of Age-Related Hearing Loss

Age-related hearing loss has mixed aetiology [7] and is likely a cumulative result of genetic and epigenetic factors [8,25,26,27] and environmental stressors [28]. Otological diseases, chronic exposure to noise, smoking, or exposure to ototoxic drugs can contribute to the development of ARHL [29,30]. Other factors include diet, gender, comorbidities, and lifestyle [20,31]. 

Reduced vascularisation in the cochlea, cumulative oxidative stress (OS), low-grade cochlear inflammation, impaired mitochondrial quality control, and mitochondrial DNA damage play a critical role in developing ARHL [8,32]. Age-related degenerative changes in the cochlea can lead to a loss of sensory hair cells and primary auditory neurons, damage to cochlear lateral wall tissues (stria vascularis and spiral ligament), and reduced vascularisation [29,33]. Post-mortem histological studies in the human cochlea have revealed sensory hair cell loss in the organ of Corti (OoC), degenerative changes in the auditory nerve, atrophy of the stria vascularis (SV), and loss of fibrocytes in the spiral ligament (SL) [20,34,35,36]. More recent studies have demonstrated a loss of auditory nerve afferent fibres in the cochlea and synapses between the inner hair cells and type I afferent fibres [37,38]. The ageing process also negatively affects the central auditory pathways [20]. Based on predominant histopathological findings and differences in pure-tone audiometric testing, Schuknetcht et al. proposed classifying ARHL into sensory, neural, strial, and cochlear conductive types [34,35,39]. Many people with ARHL likely have a mixed pathology, but in some cases, the cause of ARHL cannot be determined by histological evaluation of cochlear tissues [40]. 

At present, ARHL has not been fully reproduced in animal models. However, these models are often used to delineate human pathophysiology, as clinical studies are challenging due to cochlear localisation deep in the temporal bone, precluding histological and high-resolution imaging studies [20]. In animal studies, vascular changes include reduced capillary network and narrowing of the vascular lumen in the SV [41,42,43]. The secretory epithelium of the SV is responsible for maintaining the high potassium (K^+^) content of the endolymph and generation of the endocochlear potential (EP), which drives sensory transduction in the cochlea [44,45,46]. The SV typically deteriorates in the mid-cochlear to apical regions and is associated with reduced expression levels and activity of sodium-potassium pumps (Na-K-2Cl cotransporter NKCC1 and Na^+^, K^+^-ATPase), which leads to reduced EP [47,48,49,50,51,52,53,54]. Reduced activity of sodium-potassium pumps and decreased EP have been demonstrated in ageing gerbils raised in quiet and ageing mice [44,55]. 

The mouse is a robust and reliable mammalian model for ageing research, and the use of inbred mouse strains was instrumental in investigating the genetics of ARHL. For example, the commonly used C57BL/6 mouse strain develops progressive high-frequency hearing loss caused by a mutation of the cadherin 23 (*Cdh23*) gene, which encodes a component of the stereocilial tip-link required for gating of the mechanoelectrical transducer (MET) channel in sensory hair cells [56].

Elevated auditory thresholds in ARHL typically result from degeneration and loss of outer hair cells within the OoC. The loss of hair cells progresses from the basal turn of the cochlea (high-frequency region) to the apical turn (low-frequency region) [32]. Degenerative changes also affect synaptic networks between the inner hair cells and afferent auditory nerve fibres, which leads to reduced speech understanding in background noise [57,58,59]. 

## 4. Immunosenescence and Inflammaging

Immunosenescence is an age-dependent development of immune dysfunction that involves lymphoid organ remodelling, leading to reduced capacity to control inflammatory cytokines during and after the immune response. Immunosenescence can lead to chronic inflammation in ageing tissues, frequent infections, autoimmune diseases, and cancer due to impaired immune surveillance [21,60,61,62,63,64]. 

Inflammaging is a relatively new concept described as age-related, low-grade systemic inflammation that may not directly link to microbial infection [32,65]. Various stimuli, including cell debris, nutrients, and gut microbiota, can sustain inflammaging [66]. This sterile or pathogen-driven inflammation increases morbidity and mortality in the elderly [21,67,68]. As a result of ageing, the immune system becomes defective (immunosenescence), leading to the accumulation of unresolved inflammatory processes impacting otherwise healthy organ systems [69]. As a result, inflammaging can contribute to a spectrum of disorders such as Parkinson’s and Alzheimer’s disease, type II diabetes, and cardiovascular disease [70,71,72,73,74,75,76,77,78,79,80,81,82]. However, inflammaging is not a physiological or expected outcome of ageing; instead, a tell-tale of accelerated ageing [66]. 

Sensorineural hearing loss has also been linked with chronic inflammation [83,84,85]. Despite the historical belief that the cochlea is an immune-privileged organ [86], more recent studies have shown that the cochlea is vulnerable to systemic inflammation [22,87]. Cochlear microcirculation is controlled by tight junctions connecting vascular endothelial cells, forming the blood-labyrinth barrier (BLB) in the lateral wall [88]. The BLB plays a role in preventing pathogen infiltration, maintaining ion homeostasis, and transporting nutrients to the cochlea [89]. Pericytes and perivascular resident macrophage-like melanocytes (PVM/M) represent the second line of support for the BLB. Local inflammation activates PVM/M in the cochlea and thus increases the permeability of the BLB [90]. Furthermore, PVM/M can release proinflammatory cytokines through the tight-junction barrier [90] and increase the permeability of the BLB to the bacterial metabolite lipopolysaccharide (LPS) [88,91]. Similarly, acoustic trauma [88,92,93] and hypoxia [94] can also increase the permeability of the BLB, resulting in cochlear inflammation that predominantly affects the lateral wall tissues (SV and SL) [21,84]. It was shown that vascular cell senescence is a key factor in the breakdown of the blood-brain barrier (BBB) [95], which is physiologically and structurally equivalent to the BLB of the inner ear [22]. This suggests that vascular cell senescence may also affect the integrity and permeability of the BLB.

Inflammation has been identified in multiple preclinical and population health studies as a pathophysiological mechanism contributing to ARHL [85]. For example, in the “Hertfordshire Ageing Study”, Verschuur et al. described a progressive increase in the expression of markers associated with systemic inflammation (interleukin-6, C-reactive protein, white blood cell, and neutrophil counts) in subjects with ARHL, which correlated with the elevation in hearing thresholds [63,96]. That study concluded that low-grade inflammation is at the foundation of ARHL. In the English Longitudinal Study of Ageing, Lassale et al. also demonstrated an association between white blood cell counts and age-related hearing impairment [97]. Other studies revealed changes in the number and morphology of macrophages in the ageing cochlea [98,99]. Activated macrophages were present in the lateral wall and auditory nerve and were more abundant in the cochlear basal turn of the older donors [99]. Based on these studies, an ongoing ASPREE-HEARING study was designed to investigate the benefits of low dosages of the anti-inflammatory agent aspirin on the progression of ARHL [100]. The rationale for this study is that aspirin is an inflammation resolution mediator [101], as it decreases the levels of proinflammatory mediators, including TNF-α, IL-6, and thromboxane B2 (TXB2) [102,103,104]. The study is still recruiting participants at the time of this review. 

Preclinical studies have shown that in ageing C57BL/6J mice, the resident macrophages in the basilar membrane of the OoC change morphologically in response to sensory cell degeneration, indicating their activation [105]. This finding is consistent with the up-regulation of genes linked with immune and inflammatory responses in older murine cochleae [106]. Using next-generation sequencing, Su and collaborators [106] revealed multiple immune and inflammatory transcriptomic changes during cochlear ageing. The TNF signalling pathway, toll-like receptor signalling pathway, Jak-STAT signalling pathway, and NF-kB signalling pathway featured prominently among up-regulated genes in aged mice [106]. 

A senescence-associated secretory phenotype (SASP) is one of the possible factors contributing to inflammaging and associated changes in the central nervous system (CNS). It has been established that cells change their phenotype to senescence as a preventative measure for malignancies; however, these cells accumulate within tissues as the body ages [107]. Even though these cells are growth-arrested, they are still metabolically active and change protein expression primarily due to DNA damage [108]. SASP promotes local inflammation via the secretion of cytokines, chemokines, reactive oxygen and nitrogen species, and growth factors [107]. Proliferative cells of the CNS, such as endothelial and glial cells, can adopt SASP, leading to low-grade chronic inflammation in the ageing brain [109]. It was proposed that the permeability of the BBB might be affected by the build-up of SASP cells [95,110,111].

Despite the similarities between the BBB and the BLB, this aspect of senescence has yet to be established for the BLB and ARHL.

## 5. Chronic Inflammation

Chronic inflammation is a complex process that includes various pathophysiological mechanisms, such as OS, ionic disbalance, mitochondrial damage, and epigenetic changes, to name a few [112,113,114,115,116,117]. Immunosenescence and gut dysbiosis can also result in chronic inflammation. The natural immune response (innate and adaptive) relies on the delicate balance between proinflammatory and anti-inflammatory mediators to protect us from pathogens. However, the inflammatory process cannot be resolved when this balance is disrupted, leading to unresolved chronic inflammation [70,118]. As the body ages, cellular debris accumulates because of inefficient waste management, thus perpetuating innate immune responses that contribute to chronic inflammation [119].

Interestingly, most age-associated inflammatory disorders share similar mechanisms, mediators, and intracellular signalling pathways. Widely used biomarkers of inflammation are interleukin 6 (IL-6) [74,83,120,121,122], tumour necrosis factor-α (TNF-α) [123,124] and transforming growth factor β (TGF-β) as a pro-resolution mediator [21]. The activation of other proinflammatory cytokines (e.g., IL-1α, IL-2, IL-18) and the NF-κB signalling pathway are consistent across various tissues, including the cochlea [125,126,127,128,129,130]. Inflammaging is associated with the release of multiple inflammatory mediators, such as interleukins IL-1, IL-2, IL-6, IL-8, IL-12, IL-13, IL-15, IL-18, IL-22, IL-23, TNF-α, and interferon-γ (IFN-γ). Anti-inflammatory cytokines IL-1Ra, IL-4, IL-10, and TGF-β, are also released, while lipoxin A4 and heat shock proteins act as cytokine mediators [65,131].

### Oxidative Stress (OS) and Inflammation

OS is a well-established mechanism of sensorineural hearing loss, including ARHL [132,133]. OS is caused by the overproduction and accumulation of reactive oxygen species (ROS) [134]. ROS, produced as a by-product of cellular respiration in mitochondria, are physiologically relevant due to their role in cellular signalling and homeostasis [135]. However, multiple stressors can result in ROS overproduction and tissue accumulation [22,134]

Accumulation of ROS and other free radicals can impair DNA and break down cellular proteins and lipids, contributing to apoptotic cell death in the cochlea [85]. OS also contributes to the cellular senescence pattern in the ageing cochlea [1]. Rivas-Chacon and colleagues reported that OS and premature senescence significantly impaired cell viability in three auditory cell lines (HEI-OC1, OC-k3, and SV-k1) [1]. In addition, ROS overproduction can induce cochlear inflammation via inflammasomes [136] and thus enhance age-related sensory cell degeneration [137,138].

Inflammasomes are multiprotein oligomer components of the innate immune system, consisting of caspase 1, PYCARD (pyrin domain- or caspase activation and recruitment domain-only proteins), and pattern recognition receptors (PRRs), expressed on macrophages [139,140,141]. Inflammasome-forming PRRs include Nod-like receptor pyrin (NLRP) domain-containing proteins 1, 3, 6, 7 and 12 [142]. 

NLRP3 is associated with neurogenerative disorders [103,143], and genes encoding NLPR3 are also expressed in the cochlea [144,145]. NLRP3 is a ROS sensor, and it can bind to caspase 1 via apoptosis-associated speck-like protein containing a C-terminal caspase recruitment domain (ASC), a key adaptor molecule required for inflammatory processes [146]. The activation of caspase 1 allows for the maturation of downstream inflammatory cytokines such as interleukin-1 beta (IL-1β) and IL-18, inducing an early inflammatory response and lysis of cells by pyroptosis (a form of programmed cell death associated with inflammatory responses to microbes) [136,147,148]. NF-κB pathway activated by toll-like receptor 4 (TLR4) also up-regulates NLRP3 [149], leading to NLRP3 inflammasome priming and sterile inflammation [98]. Similarly, ROS overproduction can activate the NLRP3 inflammasome, which leads to the release of proinflammatory cytokines IL-1β and IL-18 and the activation of macrophages and microglia [98]. Based on this evidence, it was proposed that the sterile inflammation triggered by the activation of inflammasomes in the cochleae of ageing mice likely plays an important role in the pathogenesis of ARHL [136].

## 6. Association of the Gut Microbiome with Ageing and Immune System Dysfunction

Inflammaging may also result from gut dysbiosis [150]. The gut microbiome consists of the trillions of microorganisms and their genetic material in our intestinal tract. These microorganisms, mainly comprising bacteria, are critical to our health and wellbeing. However, the gut microbiome changes as we age, influencing the functions of other organs. In animal models, the permeability of intestinal barriers increases with age [150,151,152], suggesting that the leakage of gut metabolites in the elderly can potentially cause a systemic inflammatory response [22,150,153].

In young individuals, the microbiome landscape is dominated by Firmicutes and, to a slightly lesser extent, Bacteriodetes [154]. However, the elderly have a diminished population of Firmicutes and atypical Bacteriodetes/Firmicutes ratios [155] with a predominant population of Bacteriodetes [156]. Reduced microbiota diversity in aged individuals may result in the expansion of distinct groups of bacteria implicated in developing age-related type 1 diabetes mellitus, rheumatoid arthritis, and colitis [150]. This landscape can vary within certain groups from different geographical locations and is influenced by diet and genetic backgrounds [150,155,157]. For example, healthy centenarians and supercentenarians have abundant bacterial species that are considered beneficial, such as Gram-positive Bifidobacteria and non-spore-forming, anaerobic, and nonmotile Christensenella [155,157].

Bacterial metabolites and by-products can have different effects on the body. Some Firmicutes produce a short-chain fatty acid, butyrate [158]. Butyrate can mitigate age-related disorders via histone deacetylase inhibition [158] and suppression of inflammatory pathways [159,160,161], which also limits the permeability of the BBB [162]. In contrast, the microbiome in the elderly is rich in proinflammatory microbiota, such as LPS-producing Gram-negative bacteria [155,157,163], and low in butyrate producers [164]. This alteration of the gut microbiome is similar to that seen in the consumers of a high-fat diet [22].

The gut microbiota is considered a master regulator of immune homeostasis [165]. The absence of gut microbiota in germ-free mice impairs the development and maturation of the immune system, while its presence in the gut induces T regulatory cells, immunoglobulin A (IgA)-secreting B cells, Th17 cells, and type-2 lymphoid innate cells [165]. Recent studies strongly suggest that the gut microbiome can modulate the neuro-metabolic signalling pathways in the brain via a bidirectional communication with the endocrine and immune systems and thus contribute to the development of neuroinflammation and neurodegeneration [166]. It has been established that gut dysbiosis affects the permeability of the BBB by reducing the expression of tight-junction proteins, thus causing barrier dysfunction [162]. Previous studies on elderly mice with altered intestinal microbiota have shown increased levels of circulating proinflammatory cytokines compared to young mice, which was associated with anxiety-like behaviour, impaired object–place recognition memory, and social recognition [167]. In addition, gut dysbiosis associated with inflammatory bowel and celiac disease was linked to neurological manifestations [168]. 

## 7. Is Gut Dysbiosis Associated with Age-Related Hearing Loss?

In this review, we intended to capture the contribution of inflammatory processes to ARHL, and we have included age-dependent gut dysbiosis as a hypothetical link. In support, it was demonstrated that age-related dyslipidemia [169,170] and a high-fat diet [22] could lead to cochlear inflammation [171,172]. Obese mice display increased expression levels of hypoxia-induced factor 1 (HIF-1), TNF-α, NF-κB, caspase 3, poly (ADP-ribose) polymerase-1, and apoptosis-inducing factor in cochlear tissues (spiral ganglion and spiral ligament) [22,172]. Furthermore, mice fed a high-fat diet long-term showed increased immunoexpression of proinflammatory mediators in the cochlea, including intracellular adhesion molecule 1 (ICAM1), interleukin 6 receptor α (IL6Rα), and toll-like-receptor 2 (TLR2) [173]. These studies support the role of dyslipidemia and gut dysbiosis in cochlear inflammation and imply age-related changes in gut microbiota as underlying factors contributing to accelerated ARHL. 

Population health studies have demonstrated that proinflammatory food with high sugar content and consumption of beer and spirits were associated with a higher prevalence of ARHL [174]. In contrast, meta-analyses and systematic reviews of observational studies have reported lower serum concentrations of C-Reactive Protein (CRP), IL-6, and TNF-α among vegetarian and Mediterranean diet eaters compared with omnivores [175]. Differences in the diet of elderly individuals may predict their vulnerability to age-associated disorders [163]. A healthy diet rich in antioxidants is associated with a decreased risk of developing ARHL [176], while a high-fat (Western) diet can cause the accumulation of ROS in the cochlea and chronic inflammation [22,171]. These studies suggest that diet could be a modifiable factor in preventing ARHL.

**Figure 1:**
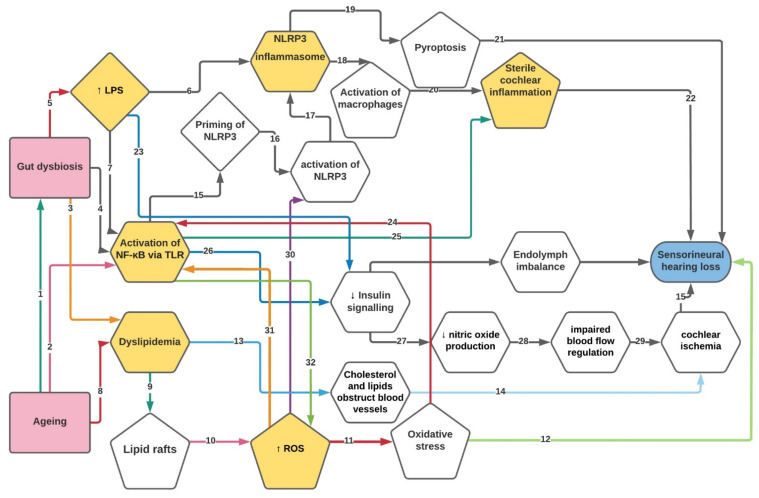
The proposed relationship between ageing, gut dysbiosis, and hearing loss. Numbers in arrows indicate a pathway described below. Abbreviations: LPS—lipopolysaccharides; ROS—reactive oxygen species; NF-κB—nuclear factor kappa-light-chain-enhancer of activated B cells; TLR—toll-like receptor; NLRP3—Nod-Like Receptor Pyrin domain-containing protein 3. Ageing is associated with developing gut dysbiosis, and it was proposed that dysbiosis is an underlying cause of age-related morbidities (**1**) [165,178]. As humans age, inflammaging results from the activation of NF-κB signalling pathways (**2**) [179]. In addition, the leaky intestinal barrier causes the release of bacterial metabolites into the bloodstream, systemic inflammation, activation of NF-κB (**4**), and ROS overproduction (**32**) [180,181,182]. Activation of NF-κB may result in reduced insulin signalling (**26**) [183,184], which can lead to sterile cochlear inflammation (**25**) [185,186], and priming of NLRP3 (**15**) [187,188,189]. Ageing (**8**) and gut dysbiosis (**3**) are both associated with dyslipidemia [22,170,190,191,192]. Dyslipidemia can lead to the narrowing of cochlear blood vessels (**13**) [193,194,195] and lipid raft formation (**9**) [196,197,198]. Lipid rafts lead to the accumulation of ROS (**10**) [197] and oxidative stress (OS) (**11**) [197,199]. OS can activate the NF-κB pathway (**24**) [200,201] and cause cochlear damage and hearing loss (**12**) [202,203,204,205,206,207,208]. The cholesterol and lipids obstructing cochlear vessels lead to cochlear ischemia (**14**), which also contributes to cochlear damage (**15**) [209,210,211,212,213,214]. Insulin resistance can lead to an electrochemical imbalance in endolymph by decreasing the expression of the sodium-potassium pump NKCC1, reducing the endocochlear potential [215,216,217]. Insulin resistance also decreases nitric oxide production (**27**) [218,219] resulting in impaired blood flow regulation (**28**) [188,220], cochlear ischemia (**29**) and eventually hearing loss (**15**) [221,222]. Gut dysbiosis can lead to increased levels of circulating lipopolysaccharide (LPS) (**5**) [223,224,225,226,227,228]. LPS is a bacterial metabolite that can activate NF-κB via toll-like receptors (TLRs) (**7**) [229,230]. LPS also affects insulin signalling (**23**) [231,232] and contributes to NLRP3 inflammasome activation [233]. After priming via the NF-κB signalling pathway (**15**), NLRP3 is activated via ROS (**30**), resulting in NLRP3 inflammasomes (**17**). NLRP3 inflammasomes can cause pyroptosis (**19**) [234], which also produces cochlear damage [32] (**21**). NLRP3 can also initiate the activation of cochlear macrophages (**18**) [32], which can increase the BLB permeability and induce sterile cochlear inflammation (**20**) [22,235,236], leading to hearing loss (**22**) [22,236].

Therefore, we postulate that a proinflammatory gut environment associated with ageing could result in a leaky gut and the translocation of pathogens and their metabolites via the systemic circulation to distant organs, including the brain and the cochlea [22,168,173]. Systemic inflammation can affect the BBB’s and BLB’s permeability and thus spread to the brain and inner ear tissues, causing local and chronic inflammation [22,177]. The proposed relationship between ageing, gut dysbiosis, and hearing loss is presented as a conceptual model in Figure 1.

## 8. Conclusions

Presbyacusis has a complex pathophysiology that is not fully understood. Here, we focus on the role of chronic, unresolved inflammation (inflammaging) and immunosenescence in the pathogenesis of ARHL. We also postulate that gut dysbiosis contributes to chronic inflammation in distant organs, including the inner ear. The concept of the gut microbiota - inner ear axis is novel, and the experimental and clinical evidence for the link between gut dysbiosis and presbyacusis is still sparse. Another limitation of this review is a potential selection bias of articles and the lack of rigorous evaluation of selected papers for validity. However, the association between gut dysbiosis and inflammaging could represent a new link to accelerated ARHL that should be explored in future studies.
